# From structural biology to designing therapy for inborn errors of metabolism

**DOI:** 10.1007/s10545-016-9923-3

**Published:** 2016-05-30

**Authors:** Wyatt W. Yue

**Affiliations:** Structural Genomics Consortium, Nuffield Department of Clinical Medicine, University of Oxford, Oxford, OX3 7DQ UK

## Abstract

At the SSIEM Symposium in Istanbul 2010, I presented an overview of protein structural approaches in the study of inborn errors of metabolism (Yue and Oppermann 2011). Five years on, the field is going strong with new protein structures, uncovered catalytic functions and novel chemical matters for metabolic enzymes, setting the stage for the next generation of drug discovery. This article aims to update on recent advances and lessons learnt on inborn errors of metabolism via the protein-centric approach, citing examples of work from my group, collaborators and co-workers that cover diverse pathways of transsulfuration, cobalamin and glycogen metabolism. Taking into consideration that many inborn errors of metabolism result in the loss of enzyme function, this presentation aims to outline three key principles that guide the design of small molecule therapy in this technically challenging field: (1) integrating structural, biochemical and cell-based data to evaluate the wide spectrum of mutation-driven enzyme defects in stability, catalysis and protein-protein interaction; (2) studying multi-domain proteins and multi-protein complexes as examples from nature, to learn how enzymes are activated by small molecules; (3) surveying different regions of the enzyme, away from its active site, that can be targeted for the design of allosteric activators and inhibitors.

## Introduction: Structure biology of metabolic enzymes is advancing

The field of structural biology has come a long way since the first protein crystal structure, that of sperm whale myoglobin, in 1958. The Protein Data Bank, a public repository of structural data determined from x-ray crystallography, nuclear magnetic resonance as well as electron microscopy (EM), has recently reached its 100,000th entry in 2014. Protein structure determination has nowadays become a streamlined process replete with technological advances that allow automation, parallelization and miniaturization of constituent steps (Su et al 2015). Examples of such pioneering development include heterologous expression systems to generate multi-component protein complexes, automated chromatography platforms for purification towards homogeneity, remedial strategies for crystallization of protein samples, as well as the implementation of high quality x-ray and electron diffraction sources worldwide.

In modern days, the term ‘structural biology’ is more appropriately coined to cover the toolkit of biophysical and biochemical, in addition to structural methods, that can probe the oligomeric assembly (e.g. size exclusion, analytical ultracentrifugation), conformational changes (e.g. spectroscopy), enzyme catalysis (e.g. Michaelis-Menton kinetics), as well as ligand/protein binding (e.g. isothermal titration calorimetry, surface plasmon resonance) features of proteins. Structural biology has therefore been instrumental in delineating the molecular functions and mechanisms of diverse target proteins, including the hundreds of human metabolic enzymes associated with inborn errors of metabolism (IEMs) (Kang and Stevens 2009; Yue and Oppermann 2011). Adopting a family-wide and pathway-wide approach in target protein selection (Osman and Edwards 2014), the Structural Genomics Consortium has to date determined nearly 50 structures of human IEM-linked metabolic enzymes (Table 1), as a first step towards developing a mechanistic understanding and therapeutic advancement of these rare genetic diseases. Our recent addition to this IEM repertoire includes structural determination of six protein players (MUT, MMAA, MCEE, MMACHC, MMADHC, MTR) involved in the different stages of the processing, trafficking and assembly of the vitamin B12 cofactor to its two destination enzymes (Froese et al 2010; Froese et al 2012; Froese et al 2015a, b). Inherited defect in each gene of this intricate B12 processing pathway gives rise to the metabolic disorders of methylmalonic aciduria and homocystinuria (Froese and Gravel 2010).

**Table 1:** Crystal structures of metabolic enzymes that are associated with inborn errors of metabolism, as determined by the SGC Oxford group of Metabolic and Rare Diseases and deposited in the public domain. Unless specified otherwise, all structures listed are of human proteins

Target name and description		Genbank ID	PDB IDs	Length	Structure region	Associated disorders (OMIM)
AASS	aminoadipate-semialdehyde synthase	13027640	*to be deposited*	926	455–926	Hyperlysinemia type I (238700)
ACACA	acetyl-CoA carboxylase alpha	38679960	2YL2, 4ASI	2383	118–654 (2YL2), 1608–2375 (4ASI)	ACACA deficiency (613933)
ACADS	acyl-CoA dehydrogenase, short chain	4557233	2VIG	412	30–412	ACADS deficiency (201470)
ACADSB	acyl-CoA dehydrogenase, short/branched chain	4501859	2JIF	432	52–432	ACADSB deficiency 2-methylbutyryl glycinuria (610006)
ACADVL	acyl-CoA dehydrogenase, very long chain	4557235	2UXW	655	72–655	ACADVL deficiency (201475)
ADA	adenosine deaminase	47078295	3IAR	363	5–363	Severe combined immunodeficiency (102700)
ALDH7A1	aldehyde dehydrogenase 7 family, member A1	4557343	2J6L	511	1–499	Pyridoxine-dependent epilepsy (266100)
CBS	cystathionine-beta-synthase	4557415	4UUU, 4COO	551	406–547 (4UUU), 1–551 (4COO)	homocystinuria due to CBS deficiency (236200)
CRYBB3	crystallin, beta B3	4758074	3QK3	211	21–199	Cataract congenital nuclear autosomal recessive type 2 (609741)
ENO3	enolase 3	153267427	2XSX	434	1–434	Glycogen storage disorder type 13 (612932)
FH	fumarate hydratase	19743875	3EO4	510	44–510	Fumarase deficiency (606812), MCUL (150800), HLRCC (605839)
FKBP14	peptidyl-prolyl cis-trans isomerase FKBP14	8923659	4DIP	211	19–140	Ehlers-Danlos syndrome types VIA and VIB (614557)
GALT	galactose-1-phosphate uridylyltransferase	22165416	5IN3	379	1–379	Classical galactosemia (230400)
GBE1	glucan (1,4-alpha-), branching enzyme 1	189458812	5CLT, 4BZY, 5CLW	702	38–700	Glycogen storage disorder type IV (232500), Adult polyglucosan body disease (263570)
GLRX5	glutaredoxin 5	42516576	2WEM, 2WUL	157	35–150	Anemia sideroblastic pyridoxine-refractory (205950)
GMDS	GDP-mannose 4,6-dehydratase	9087147	1T2A	372	23–372	Cerebellar vermis hypoplasia (602884)
GPD1L	glycerol-3-phosphate dehydrogenase 1-like protein	24307999	2PLA	351	1–349	Brugada syndrome type 2 (611777)
GYG1	glycogenin 1	12652581	3T7O,3T7O,3U2V,3U2U,3U2X,3U2T,3RMW,3RMV,3QVB,3U2W,3Q4S,3T7N,3T7M	350	1–262	Glycogen storage disease type 15 (613507)
HIBCH	3-hydroxyisobutyryl-CoA hydrolase	37594471	3BPT	386	42–386	HIBCH deficiency (250620)
HMGCS2	3-hydroxy-3-methylglutaryl-CoA synthase 2	5031751	2V4W, 2WYA	508	51–508	HMGCS deficiency (605911)
HPD	4-hydroxyphenylpyruvate dioxygenase	4504477	3ISQ	393	8–393	Tyrosinemia type 3 (276710), hawkinsinuria (140350)
HPGD	hydroxyprostaglandin dehydrogenase	1203982	2GDZ	266	3–256	Primary hypertrophic osteoathropathy (259100), isolated congenital nail clubbing (119900)
HSD17B10	hydroxyacyl-CoA dehydrogenase, type II	4758504	2O23	261	1–261	2-methyl-3-hydroxybutyryl-CoA dehydrogenase deficiency (300438), mental retardation syndromic X-linked type 10 (300220)
HSD17B4	hydroxysteroid (17-beta) dehydrogenase 4	4504505	1ZBQ	736	1–304	D-bifunctional protein deficiency (261515)
ISPD	isoprenoid synthase domain containing	157412259	4CVH	451	43–451	Muscular dystrophy-dystroglycanopathy congenital with brain and eye anomalies A7 (614643), Muscular dystrophy-dystroglycanopathy limb-girdle C7 (616052)
MAT1A	methionine adenosyltransferase I, alpha	4557737	2OBV	395	16–395	methionine adenosyltransferase deficiency (250850)
^1^MCCC1-MCCC2	3-methylcrotonoyl-CoA carboxylase complex (MCCC1* and MCCC2†)	13518228* 11545863†	*to be deposited*	725* and 563†	48–716* and 18–563†	3-methylcrotonoyl-CoA carboxylase 1 deficiency (210200)
MCEE	methylmalonyl CoA epimerase	188035928	3RMU	176	45–176	Methylmalonyl-CoA epimerase deficiency (251120)
MLYCD	malonyl-CoA decarboxylase	6912498	2YGW	454	1–451	MLYCD deficiency (248360)
MMAA	methylmalonic aciduria type A	26892295	2WWW	418	72–418	Methylmalonic aciduria type cblA (251100)
MMACHC	methylmalonic aciduria cblC type, with homocystinuria	153070822	3SOM	282	1–282	Methylmalonic aciduria cblC
^2^MMADHC	methylmalonic aciduria (cobalamin deficiency) cblD type, with homocystinuria	19527054	5A4R	296	129–296	Methylmalonic aciduria and homocystinuria type cblD (277410)
^1^MOCS2A-MOCS2B	molybdopterin synthase complex (MOCS2A* and MOCS2B†)	28631173* 4758732†	*to be deposited*	88* and 188†	9–88* and 27–179†	Molybdenum cofactor deficiency type B (252150)
MOCS2B	molybdopterin synthase catalytic subunit large subunit MOCS2B	4758732	4AP8	188	27–179	Molybdenum cofactor deficiency type B (252150)
MTR	5-methyltetrahydrofolate-homocysteine methyltransferase	4557765	4CCZ	1265	16–657	Homocystinuria-megaloblastic anemia, cblG complementation type (250940)
MUT	methylmalonyl CoA mutase	4557767	3BIC, 2XIJ, 2XIQ	750	12–750	Methylmalonic aciduria type mut (251000)
OXCT1	3-oxoacid CoA transferase	4557817	3DLX	520	40–520	SCOT deficiency (245050)
PAH	phenylalanine hydroxylase	4557819	5FII	452	19–118	Phenylketonuria (261600)
PCCA	propionyl-CoA carboxylase, alpha	65506442	2JKU	728	659–728	Propionic acidemia type I (606054)
PHGDH	phosphoglycerate dehydrogenase	23308577	2G76	533	4–315	PHGDH deficiency (601815)
PHYH	phytanoyl-CoA 2-hydroxylase	5453884	2A1X	338	31–338	Refsum disease (266500)
PTS	6-pyruvoyltetrahydropterin synthase	4506331	3I2B	145	7–145	BH4-deficient hyperphenylalaninemia type A (261640)
PYCR1	pyrroline-5-carboxylate reductase 1	24797097	2IZZ	319	1–300	Cutis laxa autosomal recessive type 2B (612940)
PYCS	pyrroline-5-carboxylate synthetase	21361368	2H5G	795	356–795	Mental retardation-joint hypermobility-skin laxity with or without metabolic abnormalities (612652)
SNX14B	sorting nexin 14 [isoform b]	24797143	4BGJ	893	505–649	Spinocerebellar ataxia, autosomal recessive, 20 (616354)
SPR	sepiapterin reductase	4507185	1Z6Z	261	1–248	Dystonia DOPA-responsive due to SPR deficiency (612716)
TGM1	transglutaminase 1	4507475	2XZZ	817	693–787	Ichthyosis lamellar type 1 (242300)
TH	tyrosine hydroxylase	88900503	2XSN	497	163–497	Segawa syndrome (605407)
TPH2	neuronal tryptophan hydroxylase	31795563	4V06	490	148–490	Attention deficit-hyperactivity disorder 7 (613003)

^1^ Structures of protein-protein complexes, with constituent subunits annotated by * and †

^2^ Structure of mouse MMADHC determined

As the field of structural biology gears towards the study of larger, more complex, and more heterogeneous biological systems, the methods of x-ray crystallography and EM, traditionally known for the respective strengths in the resolution and size of samples, will likely evolve towards the middle ground and dominate the playing field for next-generation protein structure determination. On the one hand, x-ray crystallography breaks new grounds on the size of macromolecular complexes it can resolve, as evidenced in the recent 3.6 Å crystal structure of yeast respiratory chain complex I, a mitochondrial membrane-spanning machinery comprising >14 protein subunits (Zickermann et al 2015). On the other hand, an equally impressive, 2.2 Å cryo-EM structure of an inhibitor-bound human galactosidase (Bartesaghi et al 2015) demonstrated the capability of EM to resolve ‘smaller’ complexes, but with the necessary atomic resolution to reveal ligand binding interactions.

## An integrated (structural, biochemical, computational) approach to characterize disease causing mutations for IEMs

Inborn errors of metabolism are model subjects to be studied by structural biology, due to two distinctive features. First, the predominant majority of disease-causing mutations are within the exonic region of the affected gene, rather than in the non-coding introns. Hence they are expected to affect the integrity (e.g. stability) and function (e.g. catalysis) of the encoded enzyme (Yue et al 2014), a largely globular conformation well suited for protein structure determination. Second, a large proportion of these mutations (e.g. ~65 % of all genetic variants for the metabolic disorders phenylketonuria, classical homocystinuria and ornithine transcarbamylase deficiency) are of the missense type that result in non-synonymous changes of a single amino acid, as opposed to insertion or deletion of larger peptide fragments. Missense changes are therefore more ‘subtle’ in character, context-dependent in function, and dictated by the physico-chemical properties (e.g. size and hydrophobicity) of the mutated amino acid. Structures of the wild-type and mutant metabolic enzymes determined at atomic resolution (i.e. with the details to resolve individual amino acid residues) prove useful in ascertaining the structural and molecular principles for the amino acid substitution in the local environment, as well as detecting mutation trends and hotspot regions on the protein polypeptide as a whole (Froese et al 2013; Shafqat et al 2013; Riemersma et al 2015).

Nevertheless, structural studies of disease-associated mutant proteins are often more intractable than their wild-type counterparts (Kang and Stevens 2009). This is because the mutation defects that impact on the native integrity and function of the enzyme often precludes its recombinant production in the quantity (milligrams) and quality (purity, homogeneity) that are required for the in vitro biophysical, biochemical or structural experiments. To complement this gap, a plethora of in silico prediction methods have been developed (e.g. FoldX, PolyPhen-2, SIFT and SNPs3d), adopting different algorithms of sequence, physico-chemical and structural information, to interpret the molecular effect and pathogenicity of amino acid substitutions (For reviews, see Stefl et al 2013; Studer et al 2013 and references therein). For the modern-day high-throughput pipeline in characterizing sequence variants, in silico approaches are more preferable to experimental approaches, as the latter are time-consuming and require significant resource for the design and setup.

**Fig. 1:**
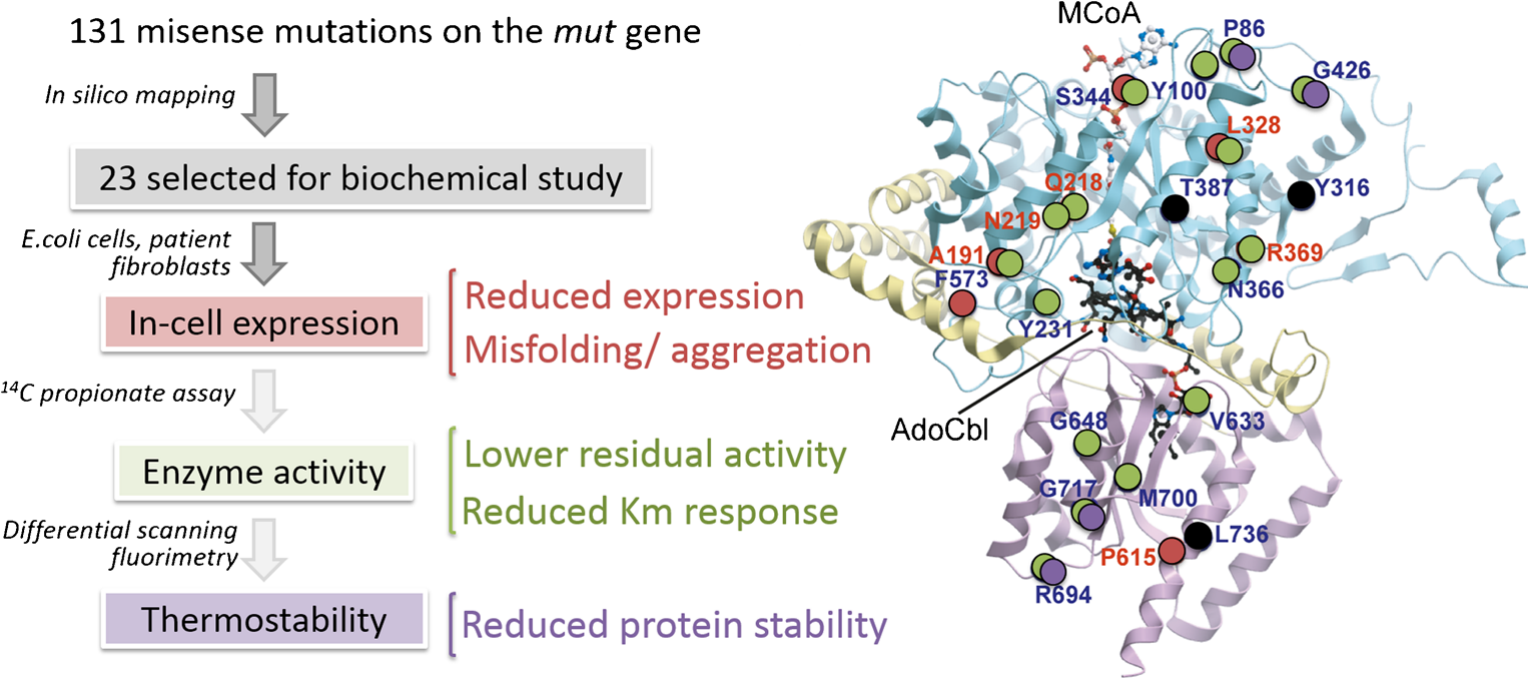
Cataloguing missense mutations in methylmalonyl-CoA mutase (MUT), applying a combination of in silico, biochemical and cellular methods to characterize protein stability and enzyme activity. Circles on the MUT crystal structure represent positions of missense mutations in this study, colour-coded to categorize their identified molecular defects

In silico methods also prove particularly useful in characterizing those metabolic disorders with broad genotypes (i.e. large numbers of disease-causing alleles), such as phenylketonuria (MIM 261600), the most common inborn errors of amino acid metabolism, where more than 400 out of 834 disease alleles annotated in the locus-specific database PAHvdb (http://www.biopku.org/home/pah.asp) are due to missense changes. A general lesson from surveying such a large catalogue of mutations (Wettstein et al 2015) is that disease-causing mutations, as opposed to the harmless variants, are often accompanied by drastic changes in the physico-chemical properties (polarity, hydrophobicity, charge, side-chain geometry) and interaction networks (hydrogen bonds, salt bridges, hydrophobic interactions) of the amino acid involved. The mutated residues are also more commonly localized within the protein core and conserved functional sites, as reflected by their strong amino acid sequence conservation. The utility of in silico prediction tools is also exemplified in its application to triage a subset of all known disease mutations as a starting point for experimental characterization of mutant proteins. This is illustrated in the example of methylmalonyl CoA mutase (MUT), one of the two destination enzymes requiring the vitamin B12 cofactor for catalysis, where to date >130 missense mutations are found on this 750-amino acid polypeptide to cause methylmalonic aciduria (MIM 251000). From a mapping of all known MUT mutations onto the protein crystal structure (Froese et al 2010), we selected 23 of them representative of diverse exonic regions, clinical phenotypes and ethnic populations, and performed a series of biochemical and cellular studies that characterize the protein stability (recombinant expression level, differential scanning fluorimetry) and enzyme catalysis (activity assay in patient cells) (Forny et al 2014) (Fig. 1). Among the different molecular defects catalogued from these mutations, we found that a subset of mutants are thermally less stable than wild-type in vitro, and can be partly rescued in vitro and in vivo by exogenous supplementation of chemical chaperones (CC) such as glycerol, proline, betaine and trimethylamine N-oxide. These CCs are low-molecular-weight osmolytes that stabilise proteins, without directly interacting with them, by preferential exclusion at the protein surface, thereby altering the thermodynamic free energy of the protein in the solvent environment (Arakawa et al 2006). Together, this study provides a proof of concept that stabilizing mutant protein could be one therapeutic strategy to rescue its defective function.

## Small molecule drug development for loss-of-function disorders is challenging

The availability of high-resolution protein structures has since facilitated greatly the process of small molecule drug development in human medicine (Yue et al 2014). Even before its high-throughput advances, structural biology already played a role in the lead optimization step of drug development, in which structures of an established therapeutic target in complex with a drug candidate can direct the chemical alterations of the drug compounds to improve their affinity, potency and selectivity. Nowadays, the timeframe of protein structure determination has become sufficiently short, hence allowing its application in other stages of the conventional drug discovery pipeline. For example, protein structures can reveal distinguishing features across different members of an enzyme/domain family to explore specificity, detect binding pockets and evaluate their ‘druggability’ (i.e. how amenable a small molecule can bind to these pockets), guide compound screening campaign using biophysical/in silico assays, and develop structure-activity relationship for a chemical series of compounds.

When considering therapeutic possibilities, inborn errors of metabolism, like other genetic diseases, can be seen as a dysregulation of dosage for the associated protein (Beaulieu et al 2012). Depending on the particular disease, or even the disease allele, a genetic lesion can theoretically lead to a higher- or lower-than-physiological level of the associated protein that signify a gain-of-function (GOF) or loss-of-function (LOF) phenotype, respectively. Our systematic study of *PAH* and *MUT* mutations, as well as of other enzymes (McCorvie and Timson 2013; Balmer et al 2014; Burda et al 2015), all conform to the general concept that IEMs are by and large LOF diseases due to pathogenic mechanisms that effect the structure (e.g. misfolding, aggregation) and function (loss of catalysis, loss of interactions) of the enzyme. This therefore poses a conceptual challenge for drug discovery, since the most intuitive therapeutic target for an IEM (i.e. the metabolic enzyme harbouring a LOF mutation itself) implies an imperative to develop an activator of the deficient or defective enzyme (Segalat 2007). While the drug development industry has more traction in GOF diseases, by means of small molecule inhibitors aimed at reducing the mRNA, protein or activity levels, the road is much less travelled for developing a therapy to activate or upregulate the levels of mRNA, protein or activity as treatment for LOF diseases. With the explosion of genomic data and disease linkage from the advent of next-generation sequencing, novel therapy design and principles are urgently required for LOF diseases (Boycott et al 2013).

## Allosteric activators as next generation pharmacological chaperones?

One emerging therapeutic approach for LOF diseases involves the use of small molecule ligands known as pharmacological chaperones (PCs) to stabilize and activate the mutant enzyme (Muntau et al 2014), with the rationale that a moderate increase in the mutant enzyme activity beyond a certain threshold level (e.g. 10 % of many lysosomal storage enzymes) could suffice to delay disease onset and ameliorate phenotypes (Suzuki et al 2009). To date, PCs have promising potentials for several IEMs, including the Fabry, Gaucher and Pompe Diseases, which have reached early-stage clinical trials (Boyd et al 2013; Parenti et al 2015); while others are gaining proof of concept (Santos-Sierra et al 2012; Jorge-Finnigan et al 2013; Makley et al 2015). These first-generation PC molecules often originate as substrate-mimetics or cofactor-mimetics of the target enzymes, and the field of structural biology has been useful in characterizing how the PC molecule interacts with the enzyme active site (Bateman et al 2011; Guce et al 2011; Torreblanca et al 2012; Suzuki et al 2014). However, active-site-directed PCs create a conundrum in which by binding to the active site, these ligands could potentially compete with the native substrate or cofactor of the target enzyme. Hence the counter-intuitive mode of action for these PCs could restrict their application to certain sub-inhibitory concentration and dosage window.

**Fig. 2:**
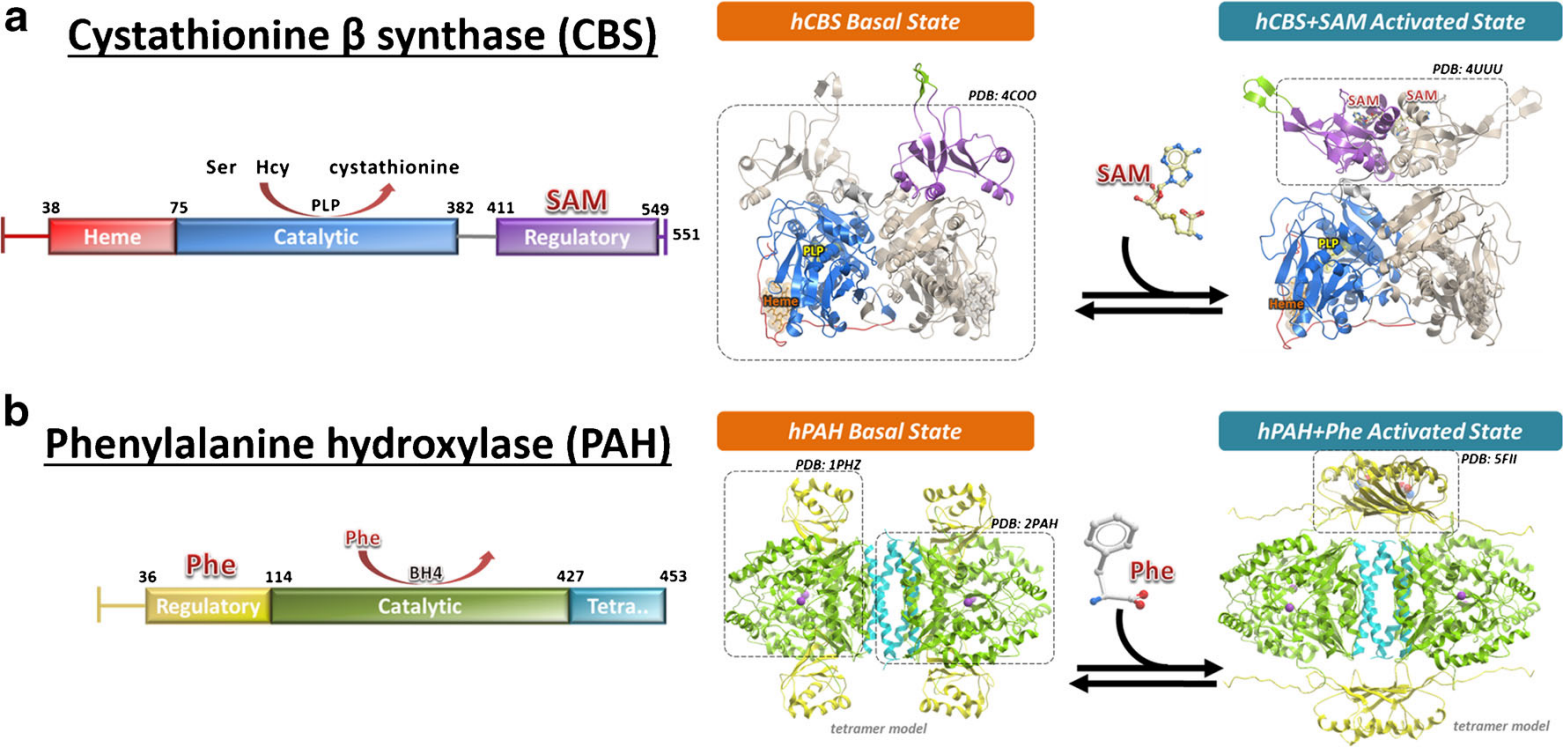
Structural biology of two multi-domain metabolic enzymes with regulatory modules. **a** Cystathionine β-synthase (CBS) is activated by *S*-adenosyl-L-methionine (SAM) which binds to the C-terminal regulatory domain (*purple*), and relieves its steric blockade of the catalytic domain (*blue*). **b** Phenylalanine hydroxylase (PAH) is activated by its own substrate phenylalanine (Phe) which binds to the N-terminal regulatory domain (*yellow*) and relieves its steric blockade of the catalytic domain (*green*). In both panels, a schematic domain organisation is shown on the *left*, and a cartoon representation of the ligand-induced conformational arrangement of regulatory domains is shown on the *right*. Structural data that are available in support of this conformational mechanism are shown in *dash-lined boxes*

We propose that the next generation of PC molecules should target an enzyme’s domains or pockets that are distant from its active site, and therefore would not compete with the enzyme’s native ligands. These allosteric, non-catalytic binding sites are often specific to an enzyme’s unique structure and function, and necessitate structural and chemical biology approaches to help identification and validation. As a first lesson to design allosteric PCs, my group has the vested interest in understanding the molecular mechanism of naturally-occurring enzymes built with a modality for ligand-binding allosteric regulation. An example for such enzyme is cystathionine-β-synthase (CBS) (Fig. 2a), which catalyzes the conversion of homocysteine to cystathionine, a reaction up-regulated by the native ligand of CBS, *S*-adenosyl-L-methionine (SAM). Inherited mutations of the *cbs* gene lead to classical homocystinuria (MIM 236200), in which protein misfolding is the major cause of malfunction among the predominant alleles. In addition to a core catalytic domain harbouring the enzyme active site, CBS contains a regulator domain at its C-terminus which binds SAM in order to activate the catalytic domain (Pey et al 2013). We have applied crystallography, biochemical and biophysical methods to explain how CBS is allosterically activated by SAM, by showing that binding of SAM to the regulatory domain results in large conformational change that relieves its steric inhibition of the enzyme catalytic domain (McCorvie et al 2014). We therefore posit that targeting the CBS regulatory domain with a small molecule could be a strategy to activate mutant CBS enzyme using the same native mechanism, thereby rescuing disease alleles with defective enzyme activity.

CBS is not a lone example of allosteric activation via a multi-domain protein architecture (Jaffe and Lawrence 2012; Jaffe et al 2013). Similarly, the enzyme phenylalanine hydroxylase (PAH), of which genetic mutations lead to PKU, is activated by its own substrate phenylalanine (Phe), postulated to bind not only to the enzyme active site in the catalytic domain, but also to a separate regulatory domain (Zhang et al 2014) (Fig. 2b). We recently provided the first structural evidence that binding of Phe to the PAH regulatory domain causes this domain to dimerize and, like CBS, relieves its steric inhibition on the catalytic domain (Patel et al 2016). We therefore believe that screening for small molecule binders in allosteric sites such as the regulatory domains of CBS and PAH could be a common principle for drug discovery of LOF inborn errors of metabolism, at least in those enzymes with allosteric sites. There are emerging studies identifying non-inhibitory ligands in allosteric sites and cryptic pockets of metabolic enzymes, either by means of high throughput screening of large compound libraries (Marugan et al 2010; Patnaik et al 2012; Porto et al 2012) or by the more chemistry-efficient small molecule fragment approach (Landon et al 2009).

## Multiple intervention avenues within a metabolic pathway

**Fig. 3:**
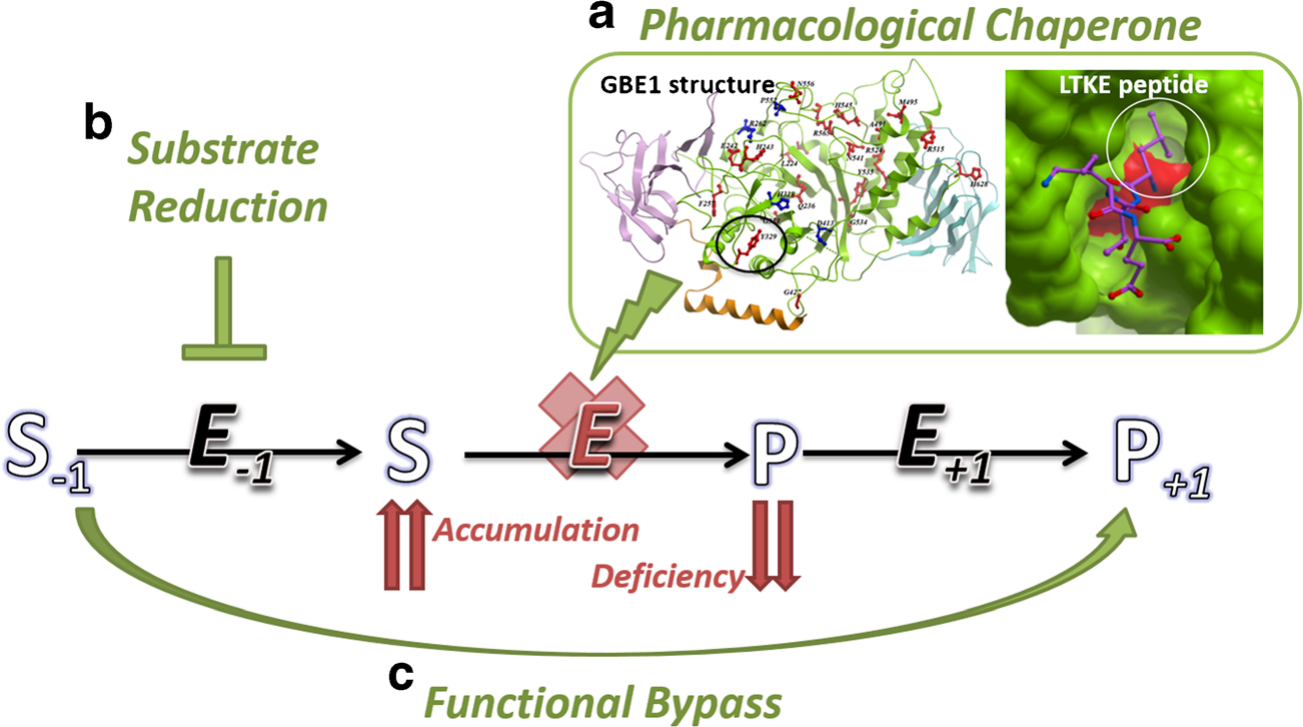
Different intervention strategies for a disease-linked enzyme within a metabolic pathway. Defects in the disease-linked enzyme (*E*) lead to the accumulation of substrate (S) and deficiency of product metabolite (P), which are connected to the upstream (*E*_*−1*_) and downstream (*E*_*+1*_) enzymes of the metabolic pathway. The different points of intervention include **a** pharmacological chaperones, **b** substrate reduction therapy and **c** functional bypass. An example of pharmacological chaperone development is shown in Fig. 3a inset, for the glycogen branching enzyme GBE1, where the prevalent disease mutation p.Y329S (*circled*) creates a cavity at the protein surface. A synthetic peptide LTKE (*purple sticks*) is designed to fill this void and stabilize the mutant protein

When determining the appropriate therapeutic targets for a metabolic disorder, it is often beneficial to look at the different reaction steps around the affected enzyme of a metabolic pathway, in order to maximize intervention possibilities (Fig. 3). As an example, normal glycogen synthesis is catalysed by the concerted action of three enzymes in humans: glycogenin (GYG), glycogen synthase (GYS) and branching enzyme (GBE). Andersen disease (MIM 232500) and adult polyglucosan body disease (APBD; MIM 263570) caused by GBE mutations, as well as Lafora disease due to mutations in malin or laforin proteins (MIM 254780), are all neurological diseases sharing a common neuropathology of malformed glycogen (‘polyglucosan’) accumulation, although their genetic defects affect different aspects of glycogen metabolism. We have recently applied structural biology to characterize GBE disease-causing mutations, in particular the prevalent allele causing APBD, p.Y329S. A homology model of the GBE mutant, based on the experimental wild-type crystal structure (Froese et al 2015), shows that the Tyr329-to-Ser substitution creates a cavity at the protein surface away from the active site, and results in lower protein expression in vitro and in vivo, and reduced protein stability. We reasoned that a small molecule specifically targeting this surface-accessible cavity could function as a ‘molecular strut’ to stabilize the local defect of this mutant enzyme. As proof of therapeutic principle, a tetra-peptide (Leu-Thr-Lys-Glu) designed computationally to fill this cavity can be taken up in patient cells, binds specifically to mutant GBE protein and rescues mutant enzyme activity to a moderate level (Fig. 3a).

The peptide approach above can be considered an example of pharmacological chaperone that is targeted directly at the site of mutation defect, distant from the enzyme active site. An alternative therapeutic avenue, however, can be based on substrate inhibition, with the rationale that the same biosynthesis enzymes for normal glycogen production are also responsible for polyglucosan formation. Recently, a gene knockdown of muscle glycogen synthase (GYS1) eliminated polyglucosan formation and restored neurological functions in a mouse model of Lafora disease (Pederson et al 2013), as well as a neuronal model for APBD (Kakhlon et al 2013). These clinical findings are further supported by the natural existence of very rare GYS mutations which lead to its enzyme deficiency (MIM 611556) with mild phenotypes in humans. Together, they provide proof of principle that downregulation of glycogen synthesis eliminates neurological abnormalities due to polyglucosan formation, and lend support to the inhibition of GYS1 enzyme activity, or its interaction with the functional partner GYG necessary for glycogen synthesis, as potential molecular therapy for Lafora and Andersen diseases, as well as for the lysosomal storage disorder Pombe (GAA mutations) whose hallmark is muscle glycogen accumulation. The inhibition approach is particularly attractive to the drug development industry, and can be considered as an example of ‘substrate reduction therapy’ (Fig. 3b) where there is precedence in IEMs (e.g. Miglustat for the treatment of Gaucher disease (Platt and Jeyakumar 2008)) to reduce the consequence of toxic metabolite accumulation due to defective enzymes (Schiffmann 2015). We anticipate that progress in the structural biology of GYS and GYG (Chaikuad et al 2011; Zeqiraj et al 2014) should pave the next step forward in developing novel inhibitors for these enzymes. This example therefore illustrates the principle of targeting different components of a metabolic pathway for the treatment of a genetic disease. As we enter the post-genomic era from the advent of next generation sequencing, with emerging new diseases (Ebrahimi-Fakhari et al 2016) and unmet medical need, there is an urgent call for novel, ‘imaginative’ routes of therapeutic intervention to tackle IEMs, and rare diseases as a whole. To this end, a recent example of functionally bypassing a deficient protein by a suppressor mutation (Yoon et al 2014) may represent one of many future therapeutic approaches aimed at activating an alternative gene, isoform or enzymatic mechanism (Fig. 3c) to functionally compensate for the defective gene and product.
